# Drinking Patterns and Problems Among African-Americans: Recent Findings

**Published:** 1998

**Authors:** Rhonda Jones-Webb

**Affiliations:** Rhonda Jones-Webb, Dr.P.H., is associate professor in the Division of Epidemiology, School of Public Health, University of Minnesota, Minneapolis, Minnesota

**Keywords:** AOD use pattern, African American, AOD abstinence, AOD dependence, AOD associated consequences, heavy AOD use, demographic characteristics, cause of AODU (alcohol and other drug use), age, socioeconomic status, spirituality and religion, coping skills, white American, comparative study, literature review

## Abstract

The findings of recent research on drinking patterns and problems among African-Americans can be summarized as follows: (1) African-Americans report higher abstention rates than do whites; (2) African-Americans and whites report similar levels of frequent heavy drinking; (3) rates of heavy drinking have not declined at the same rate among African-American men and women as among white men; and (4) variables such as age, social class, church attendance, drinking norms, and avoidance coping may be important in understanding differences in drinking and drinking problem rates among African-Americans and whites. Limitations of the research are described and suggestions are made for possible directions for future research.

Alcohol studies on African-Americans make up a small but growing body of research. The limited research that does exist has focused on a number of issues, including patterns and determinants of drinking and drinking problems. This article reviews recent studies examining the drinking patterns and problems of adult African-Americans. Studies of African-Americans have, for the most part, involved comparisons of drinking patterns and drinking problems among African-American and white populations. This review therefore focuses primarily on studies of that type. After discussing drinking patterns and problems among African-Americans, the article describes some of the possible determinants of drinking patterns in that population.

## Drinking Patterns

The term “drinking patterns” refers to various styles of drinking, such as abstention or heavy drinking. Categories of drinking patterns frequently are based on indexes of quantity and frequency or on assessments of the volume of alcohol consumed by a person in a given period of time. Unless otherwise noted, terms describing drinking patterns are based on the definitions used in the studies being discussed.

National surveys examining the prevalence of drinking in African-American populations indicate that African-Americans report lower rates of alcohol use than whites. In 1997, according to the National Household Survey on Drug Abuse, 53 percent of African-Americans and 68 percent of whites reported drinking in the past year (U.S. Department of Health and Human Services 1998). The differences were most striking between African-American and white women (48 percent and 64 percent, respectively). Similarly, in 1992 the National Alcohol Survey found an abstention rate of 51 percent among African-American women and 35 percent among African-American men, compared with a rate of 36 percent among white women and 28 percent among white men (Caetano and Kaskutas 1995). The National Longitudinal Alcohol Epidemiologic Survey also found lower rates of alcohol use among African-Americans than among whites (Dawson et al. 1995). In addition, surveys conducted at the community level (e.g., Darrow et al. 1992) comparing rates of drinking in African-American and white populations have reported lower rates of alcohol use in African-Americans than in whites.

Heavy drinking increases the risk of drinking problems; researchers therefore often focus on heavy drinking when comparing African-American and white drinkers. Data from the National Alcohol Survey (Caetano and Kaskutas 1995) show that although African-Americans report higher rates of abstention than whites, the two groups report similar levels of frequent heavy drinking (i.e., consuming five or more drinks at one sitting at least once per week) (see table 1). In the 1992 National Alcohol Survey, rates of frequent heavy drinking^1^ were 15 percent for African-American men and 12 percent for white men, compared with 3 percent for African-American women and 5 percent for white women (Caetano and Kaskutas 1995). Rates of heavy drinking may vary across studies, however. For example, in one study of African-American and white women in Erie County, New York, Darrow and colleagues (1992) found slightly higher rates of heavy drinking (12 percent among African-American women and 17 percent among white women) than did the National Alcohol Survey. Differences in survey findings may be attributable to regional differences, survey methodology, and other factors.

Panel studies^2^ of African-Americans are rare (Bachman et al. 1997; Caetano and Kaskutas 1995). Caetano and Kaskutas examined changes in drinking patterns among a large sample of African-Americans and whites from 1984 to 1992. The researchers found that abstention increased significantly among all the subjects, both African-American and white (see table 1). Although frequent heavy drinking decreased significantly among white men, it remained stable among African-American men and women as well as among white women during that same period.

## Drinking Problems

Two of the most widely studied indicators of drinking problems include drinking consequences and alcohol dependence symptoms. Drinking consequences are concrete problems that arise in different areas of a person’s life because of drinking (e.g., financial difficulties, illness, troubles with relationships, and work-related and legal problems). Alcohol dependence symptoms refer to a set of behaviors and experiences associated with alcoholism or addiction, such as withdrawal and blackouts (Hilton 1991). Some studies (Grant 1997; Herd 1994*a*) have found that African-Americans report significantly higher numbers of drinking consequences and alcohol dependence symptoms than do whites in the past 12 months, whereas other studies (Kandel et al. 1997; Lozina et al. 1995) have reported no significant differences between the two groups.

Longitudinal surveys have focused on changes in drinking-problem indicators and alcohol-related mortality among African-Americans and whites. Rates of alcohol dependence symptoms and drinking consequences remained stable from 1984 to 1995 among African-American and white men and women (Caetano and Clark 1998) (see table 2, p. 262). Another study found that specific alcohol-related mortality rates (controlled for age) decreased among African-Americans and whites from 1979 to 1989; however, rates of alcohol-related mortality were consistently higher among African-Americans than among whites over the 10-year period (Stinson et al. 1993). Thus, although African-Americans and whites report similar rates of frequent heavy drinking, African-Americans are more likely to die of alcohol-related illnesses and injuries, such as cirrhosis of the liver and alcohol-related car crashes. Some evidence (Caetano and Kaskutas 1995) indicates that compared with whites, African-Americans may have longer heavy drinking “careers,” which may account for the disparity in alcohol-related illness.

Some of the most promising studies on drinking problems have examined the relationships between race, social class, and drinking problems. Studies in this area have focused on two central hypotheses. The first hypothesis is that adjusting for social class may eliminate racial and ethnic differences in drinking problem rates. Herd (1994*a*) tested this hypothesis but found no support for it. Rather, after adjusting for income, education, occupation, and employment status, she found that African-American men reported significantly greater numbers of drinking consequences than did white men. The second hypothesis is that racial differences in rates of drinking problems exist but are limited to certain social classes. Empirical support exists for the latter hypothesis. Using data from a statewide survey of New York, Barr and colleagues (1993) found that African-American men with relatively low incomes were significantly more likely than their white counterparts to report high rates of alcohol dependence symptoms; the reverse was true for African-American and white men with relatively high incomes.

To summarize, studies comparing self-reported drinking problems in African-American and white populations have yielded mixed results. Some studies have shown that African-Americans report more drinking problems than do whites, whereas other studies have reported no significant differences between the two groups. Higher social class appears to be a protective factor for African-Americans against the effects of race on drinking problems. Continuing research is needed to understand the effects of social class status on drinking problems among African-Americans.

## Determinants of Drinking and Drinking Problems

For the most part, research on drinking patterns and drinking problems in African-American populations has not been based on theory. Nevertheless, etiological studies of drinking patterns and drinking problems in African-American populations have focused on a wide range of demographic, biological, environmental, and behavioral variables.

### Demographic Factors

Sex, age, income, education, and employment status have been shown to be related to drinking patterns and drinking problems in both African-American and white populations (Herd 1994*a*, 1997). Important differences exist in how certain demographic variables are correlated with drinking patterns and problems in the two groups. For example, frequent heavy drinking is associated with youth and high income in whites but with older age and low income in African-Americans (Herd 1990). Likewise, church attendance has been found to be positively related to abstinence and negatively related to heavy drinking among African-Americans but not among whites (Darrow et al. 1992).

### Biological Factors

In addition to demographic variables, studies also have examined whether a family history of alcohol problems contributes to drinking problems. Two of the studies reviewed for this article reported—and many researchers agree—that a family history of drinking problems is an important correlate of drinking behavior (Darrow et al. 1992; Lozina et al. 1995). People with a family history of alcohol problems may have different drinking patterns from those who do not have such a family history; in addition, those drinking patterns may lead to greater drinking problems.

### Environmental Factors

Studies have examined the influence of stress, parental and peer attitudes about drinking, drinking norms, and drinking contexts (i.e., physical setting and situation) on drinking patterns and problems among African-Americans. Taylor and Jackson (1990) tested a model predicting the level of alcohol consumption in a sample of 289 African-American women. They found that stress had a direct and positive effect on alcohol consumption (i.e., as the number of stressful events increased, alcohol consumption also increased). They also found that stress indirectly affected alcohol consumption through physical health problems (i.e., as stress produced physical symptoms, drinking decreased).

To see how stress influenced drinking, Cooper and colleagues (1992) tested a stress model in a sample of 1,933 African-American and white adults. The researchers found that stress more strongly influenced alcohol use and drinking problems among the African-American subjects who coped with stress primarily through avoidance (i.e., high-avoidance-coping African-Americans) than among high-avoidance-coping white subjects.

Herd (1994*b*) examined the relationships between parental drinking attitudes, drinking norms, and drinking patterns among African-American and white women. She found that parental drinking attitudes indirectly influenced drinking patterns through their effects on drinking norms. If respondents’ parents had liberal drinking attitudes, respondents also were more likely to have such attitudes. Among respondents, liberal attitudes were associated with more frequent drinking. Drinking norms have been found to be related to drinking problems in long-term studies as well. Jones-Webb and colleagues (1997) examined the relationships between normative attitudes and changes in drinking problems in African-American and white men and women between 1984 and 1992. Among men who became more liberal in their nonsocial drinking norms,^3^ drinking consequences increased for African-Americans but not for whites.

Herd and Grube (1993) investigated the relationships between drinking contexts and drinking problems in African-American and white women. The purpose of the study was to determine whether African-American and white women differed in how often they drank in various social settings and whether drinking in different contexts was independently related to drinking problems. The study analyzed the effects of three drinking contexts on drinking problems: drinking in the home; drinking in social settings, such as restaurants and bars; and drinking in outdoor public areas, such as street corners and parks. Herd and Grube found that race was not directly related to drinking contexts but that drinking contexts were directly related to drinking problems.

### Behavioral Factors

Studies have consistently found a positive relationship between drinking and drinking problems—that is, as drinking increases, drinking-related problems increase (Herd 1994*a*; Jones-Webb et al. 1995). Increases in alcohol consumption may have greater long-term consequences for white than for African-American men. In their study of drinking patterns between 1984 and 1992, Jones-Webb and colleagues (1997) studied predictors of increases in alcohol-related problems in African-American and white men. Among African-American and white men who increased their alcohol consumption, white men were more likely than were African-American men to report increases in drinking consequences.

## Summary

Research regarding drinking patterns and drinking problems in African-Americans can be summarized as follows:

- African-Americans report higher abstention rates than do whites.
- African-Americans and whites report similar levels of frequent heavy drinking.
- Rates of heavy drinking have not declined at the same rate among African-American men and women as among white men.
- Variables such as age, social class, church attendance, drinking norms, and coping behaviors may be important in understanding differences in drinking and drinking problem rates among African-Americans and whites.

Studies examining the determinants of drinking patterns and problems suggest that demographic and psychological variables may be important in understanding the differences in drinking patterns and drinking problems among African-American and white men and women. Despite the wide range of variables that have been studied, however, notable gaps exist in the research. None of the studies reviewed examined the effects of illicit drugs and tobacco use, the concentration of stores selling alcohol in a given area (i.e., alcohol outlet density), or exposure to alcohol through the mass media on drinking patterns and drinking problems. The scant research on alcohol outlet density and media exposure is surprising, given the large number of alcohol outlets in many African-American inner cities and efforts by the alcohol industry to target African-Americans (see the article by Alaniz, pp. 286–289). Future studies should focus on those variables. In addition, few studies have included other ethnic populations, such as Asians and American Indians; those studies are needed to investigate the reasons behind the different trends in heavy drinking patterns for different ethnic groups.

Continuing research is needed on the drinking patterns and drinking problems of African-Americans to address current gaps in knowledge. To ensure that interventions and policies to reduce drinking problems in African-American populations are guided by sound research, future studies will need to be theory based; use a wide range of study designs and variables; and include comparisons with other ethnic groups, such as Hispanics, Asians, and Native Americans. Panel studies also are needed to better understand changes in drinking patterns of African-Americans over time.

## Figures and Tables

**Table 1:** Drinking Patterns Among Whites and Blacks, 1984 and 1992

	Year			
	
	1984		1992	
		
Drinking Pattern^1^	Whites (%)	Blacks (%)	Whites (%)	Blacks (%)
**Men**				
Abstainer	23	29	28	35
Infrequent	13	13	9	6
Less frequent	16	12	21	19
Frequent	27	30	29	25
Frequent heavy	19	16	12	15
**Women**				
Abstainer	31	46	36	51
Infrequent	23	18	22	24
Less frequent	19	19	24	12
Frequent	23	13	15	8
Frequent heavy	4	4	3	5

1 Abstainer = drinks less than once per year or has never consumed alcoholic beverages; infrequent = drinks less than once per month but at least once per year and may or may not drink five drinks at one sitting; less frequent = drinks one to three times per month and may or may not drink five or more drinks at one sitting; frequent = drinks once per week or more and may or may not consume five or more drinks in one sitting; frequent heavy = drinks once per week or more and has five or more drinks at one sitting at least once per week.

SOURCE: Caetano and Kaskutas 1995.

**Table 2:** Prevalence of Alcohol Problems Among White and Black Men and Women, 1984 and 1995

	Year			
	
	1984		1995	
		
Number of Problems^1^	Whites (%)	Blacks (%)	Whites (%)	Blacks (%)
**Men**				
0	75	73	78	75
1	8	7	7	9
2	5	4	4	3
3+	12	16	11	13
**Women**				
0	86	90	88	89
1	6	5	5	4
2	3	2	3	2
3+	5	3	4	4

1 Problems were salience of drinking, impaired control, withdrawal, relief drinking, tolerance, binge drinking, belligerence, accidents, health-related problems, work-related problems, financial problems, problems with the police, problems with spouse, and problems with persons other than spouse.

SOURCE: Caetano and Clark 1998.

## Footnotes

1 The study defined a frequent heavy drinker as a person who consumes at least five drinks at one sitting at least once per week.

2 Panel studies follow the same subjects over time and are important in analyzing changes in drinking patterns.

3 Nonsocial drinking norms are beliefs regarding the appropriateness of drinking in nonsocial settings, such as drinking while taking care of a child.
